# Evaluating the Performance of Sputum-Based Targeted Sequencing Against Mycobacterial Whole-Genome Sequencing in Predicting Tuberculosis Drug Resistance

**DOI:** 10.3390/ijms27125392

**Published:** 2026-06-15

**Authors:** Lili Tian, Nenhan Wang, Xiaowei Dai, Shuangshuang Chen, Hao Chen, Jie Li, Chuanyou Li, Hongtai Zhang

**Affiliations:** 1 Beijing Center for Disease Prevention and Control, Beijing 100035, China; 2 School of Public Health, Capital Medical University, Beijing 100069, China

**Keywords:** tuberculosis, targeted sequencing, whole-genome sequencing, resistance

## Abstract

Direct sputum targeted next-generation sequencing (tNGS) offers rapid resistance profiling without culture, but its concordance with isolate-based whole-genome sequencing (WGS) and phenotypic drug susceptibility testing (pDST) remains unclear. We compared tNGS (direct sputum) and WGS (cultured isolates) for 14 drugs’ resistance prediction in 68 culture-positive tuberculosis specimens, using pDST as reference. Performance for lineage concordance was also assessed. tNGS showed the highest rifampicin (RIF) sensitivity (90.9%) but the lowest specificity (65.2%); WGS achieved the best overall agreement (86.8%). For isoniazid, tNGS sensitivity was 82.6% vs. WGS 78.3%, but WGS specificity was higher (91.1% vs. 75.6%). tNGS outperformed WGS for ethambutol (EMB) sensitivity (80.0% vs. 40.0%). Both methods performed poorly for pyrazinamide (PZA) (agreement ~40%). Among 68 specimens, 51.5% had fully concordant resistance profiles; tNGS-only variants outnumbered WGS-only variants 2:1. Crucially, tNGS and WGS on the same 24 cultured isolates yielded identical results, proving that discrepancies arise from culture-driven clonal selection, not technical differences. Direct sputum tNGS suggests broader within-host resistance diversity that may be missed by culture, whereas WGS offers superior specificity. The two methods are complementary, with culture bias being the primary source of discordance.

## 1. Introduction

Drug-resistant tuberculosis (DR-TB), particularly multidrug-resistant (MDR) and rifampicin-resistant (RR) TB, remains a major global public health threat. In 2023 alone, an estimated 1.25 million people died from TB worldwide, and more than 400,000 new cases developed that were resistant to rifampicin (RIF), the most effective first-line drug [1]. The accurate and rapid detection of *Mycobacterium tuberculosis* (MTB) drug susceptibility is critical for guiding effective treatment regimens and curbing the spread of drug-resistant strains. Traditional phenotypic drug susceptibility testing (pDST) has long served as the diagnostic reference standard, but it relies on mycobacterial culture—a slow process typically taking weeks to months—thus failing to meet the urgent clinical need for timely therapeutic decisions [2].

Molecular diagnostic technologies have advanced substantially. Xpert^®^ *Mycobacterium tuberculosis*/rifampicin (Xpert MTB/RIF), a widely deployed rapid molecular test, can detect both MTB and RR directly from sputum, its drug testing coverage [3]. Whole-genome sequencing (WGS) provides comprehensive information on drug resistance mutations and has been extensively adopted for resistance surveillance and epidemiological investigations [4,5]. However, WGS conventionally requires DNA extraction from cultured isolates, which not only prolongs turnaround times but, more critically, may introduce clonal selection bias during in vitro culture. The serial subculture of MTB results in the significant dynamic loss of minor-variant resistant subpopulations across all resistance-determining regions, with 13% of isolates transitioning from resistant to susceptible for at least one drug [6]. A selective expansion of dominant subclones may lead to the loss or suppression of low-abundance resistant subpopulations originally present in the clinical specimen, thereby underestimating true within-host resistance diversity [7].

To overcome the limitations of culture dependence, targeted next-generation sequencing (tNGS) applied directly to sputum specimens has emerged as a promising alternative. By selectively enriching specific drug resistance-associated genes, direct sputum tNGS can generate comprehensive resistance profiles without prior culture, offering reduced turnaround time (typically 3–4 days) and greater cost-effectiveness compared with WGS [8,9]. Several recent studies have explored its feasibility. For example, a systematic review reported high concordance between tNGS and phenotypic DST for multiple drugs [10], and direct sputum tNGS was shown to be feasible even in smear-positive specimens [11]. However, direct comparisons with both WGS and pDST across a comprehensive panel of first- and second-line drugs remain limited.

Despite these encouraging findings, the consistency between direct sputum tNGS and isolate-based WGS—against the reference standard of pDST—has not been systematically evaluated. More importantly, when the two molecular approaches yield discordant results, it remains unknown whether such discrepancies arise from inherent technical differences between sequencing platforms (targeted capture versus whole-genome) or from genuine biological mismatches driven by the culture step itself. Previous work using deep amplicon sequencing provided evidence that mycobacterial culture induces clonal selection, with “gain-and-loss” of resistant variants observed when comparing pre-culture and post-culture specimens [6,12]. However, direct side-by-side evidence comparing tNGS and WGS on identical DNA materials has been lacking, leaving the source of discordance unresolved.

To address these gaps, the present study systematically compared direct sputum tNGS, culture-isolate WGS, and Xpert MTB/RIF for the prediction of RR, and extended the comparison to isoniazid (INH), ethambutol (EMB), pyrazinamide (PZA), fluoroquinolones (FQs), and a range of second-line drugs in 68 smear-positive, culture-positive TB specimens, using pDST as the reference. We assessed the diagnostic accuracy, overall agreement, and discordant mutation profiles between the two methods. Critically, we performed both tNGS and WGS on the same 24 cultured isolates to definitively isolate the effect of technology from the effect of culture. This design clarified the pros and cons of direct sputum tNGS and isolate-based WGS for DR-TB diagnosis and verified that culture bias, not technical differences, dominates inter-assay discordance. The findings in this study are intended to inform the optimal placement of each technology within the evolving World Health Organization (WHO)-endorsed tiered diagnostic framework for DR-TB [13].

## 2. Results

### 2.1. Comparison of WGS (On Cultured Isolates) Versus Direct Sputum tNGS and Xpert MTB/RIF for RR Prediction

A total of 68 sputum specimens from patients with pulmonary tuberculosis, all culture-positive for MTB, were included in this study. Direct tNGS and Xpert MTB/RIF performed on raw sputum specimens (without prior culture) were positive for all 68 specimens. Using culture as the gold standard, both molecular methods achieved 100% sensitivity for MTB detection.

With pDST of post-culture isolates as the reference standard, we compared the diagnostic accuracy of tNGS (direct sputum), WGS performed on cultured isolates, and Xpert MTB/RIF (direct sputum) for predicting RR (Table 1). tNGS showed the highest sensitivity (90.91%, 95% CI: 69.38–98.41), followed by Xpert MTB/RIF and WGS, both at 86.36% (95% CI: 64.04–96.41). Regarding specificity, WGS was the highest (86.96%, 95% CI: 73.05–94.58), Xpert MTB/RIF was intermediate (80.43%, 95% CI: 65.62–90.14), and tNGS was the lowest (65.22%, 95% CI: 49.68–78.23). The overall diagnostic agreement was best for WGS (86.76%), followed by Xpert MTB/RIF (82.35%) and tNGS (73.53%). The Kappa values were 0.708 for WGS, 0.624 for Xpert MTB/RIF, and 0.481 for tNGS. The χ^2^ values were 34.413 for WGS, 27.461 for Xpert MTB/RIF, and 18.818 for tNGS. These findings indicate that WGS based on cultured isolates achieves better concordance with pDST for RR prediction than direct sputum tNGS.

### 2.2. Comparison of WGS (On Cultured Isolates) Versus Direct Sputum tNGS for Predicting Resistance to Other Anti-Tuberculosis Drugs

Using pDST of post-culture isolates as the reference standard, we assessed the performance of WGS (performed on cultured isolates) and tNGS (performed directly on sputum) for predicting resistance to other first-line drugs: INH, EMB, and PZA. Detailed results are summarized in Table 2.

INH: tNGS showed a sensitivity of 82.61% (95% CI: 60.45–94.28), slightly higher than that of WGS (78.26%, 95% CI: 55.79–91.71); however, the specificity of WGS (91.11%, 95% CI: 77.87–97.11) was significantly higher than that of tNGS (75.56%, 95% CI: 60.14–86.61). Consequently, the overall diagnostic agreement of WGS (86.76%) was superior to that of tNGS (77.94%). In this cohort, WGS produced four false-positive resistance calls, whereas tNGS produced 11.

EMB: tNGS exhibited a marked advantage in sensitivity (80.00%, 95% CI: 29.88–98.95) compared with WGS (40.00%, 95% CI: 7.26–82.96), while specificities were comparable (84.13% vs. 85.71%). The high false-negative rate of WGS (three resistant isolates called susceptible) suggests that culture-based sequencing may fail to detect certain ethambutol resistance mutations that are captured by direct sputum tNGS. Notably, the number of phenotypically EMB-resistant specimens was small (*n* = 5), resulting in wide confidence intervals; therefore, caution is warranted in interpretation.

PZA: Both methods performed poorly against PZA. The sensitivities of tNGS and WGS were 9.30% (95% CI: 3.02–23.05) and 13.95% (95% CI: 5.80–28.63), respectively, and the diagnostic agreements were 38.24% and 41.18%, respectively. This poor performance may reflect the diversity of mutations in *pncA* (the main resistance-determining gene for pyrazinamide), for which genotype–phenotype correlations are notoriously challenging. Hence, pDST remains indispensable for PZA susceptibility assessment in clinical practice.

For second-line and other drugs, the comparison between tNGS and WGS showed different patterns. For BDQ, DLM, LZD, AMK, KAN, CAP, and PAS, tNGS (direct sputum) and WGS (on cultured isolates) were completely concordant with respect to mutation detection and resistance prediction calls. However, when pDST of cultured isolates was used as the reference, diagnostic agreement varied considerably: BDQ 97.06%, LZD and AMK 94.12%, KAN 89.71%, CAP 83.82%, DLM 69.12%, and PAS only 52.94%. The low agreement for PAS highlights the challenges in genotypic susceptibility testing for this drug, as its resistance mutations are incompletely characterized, and a high proportion of phenotypically resistant isolates lack known resistance determinants.

For MFX and LFX, WGS (on cultured isolates) demonstrated superior sensitivity to direct sputum tNGS: for MFX, WGS 70.83% (95% CI: 48.75–86.56) vs. tNGS 50.00% (95% CI: 29.65–70.35); for LFX, WGS 76.47% (95% CI: 49.76–92.18) vs. tNGS 58.82% (95% CI: 33.45–80.57). In addition, WGS achieved higher specificity and overall agreement.

For STM, tNGS showed higher sensitivity (80.00% vs. 76.00%), whereas WGS exhibited superior specificity (95.35% vs. 81.40%), a pattern similar to that observed for RIF and INH.

Direct sputum tNGS demonstrated higher sensitivity for detecting resistance mutations to INH, EMB, and STM, enabling the capture of resistant subpopulations that may be lost during culture, thereby reflecting a more authentic in vivo resistance profile. In contrast, WGS based on cultured isolates generally showed better specificity and overall diagnostic agreement, particularly for INH and FQs, with higher concordance with pDST. For drugs such as PZA, PAS, and DLM, both molecular methods yielded low agreement with pDST, suggesting complex resistance mechanisms or an incomplete coverage of known resistance genes; thus, pDST remains essential for accurate susceptibility assessment.

### 2.3. Discrepancies in Resistance-Associated Mutation Detection Between Direct Sputum tNGS and WGS on Cultured Isolates

Among the 68 specimens, 35 (51.47%) showed completely concordant resistance profiles between tNGS (direct sputum) and WGS (on cultured isolates) (Table S1). The distribution of discordant profiles was as follows: 10 specimens (14.71%) differed for one drug, 7 (10.29%) for two drugs, 9 (13.24%) for three drugs, and 7 (10.29%) for more than three drugs.

Discordances were mainly attributable to differences in variant detection within the tNGS target regions. A total of 74 discordant events were identified: 49 variants were detected only by tNGS, 25 only by WGS, and a further 13 discordant events were due to differences in the positions of resistance-associated mutations called by the two methods.

Notably, the ratio of tNGS-only discordant events to WGS-only discordant events was approximately 2:1. This observation suggests that sputum culture may preferentially expand a particular subclone of M. tuberculosis, and therefore the mutation profile obtained from post-culture isolates (WGS) may not fully represent the actual resistance spectrum of the infecting bacterial population.

### 2.4. Genotype Concordance Between Direct Sputum tNGS and WGS on Cultured Isolates

**Figure 1:**
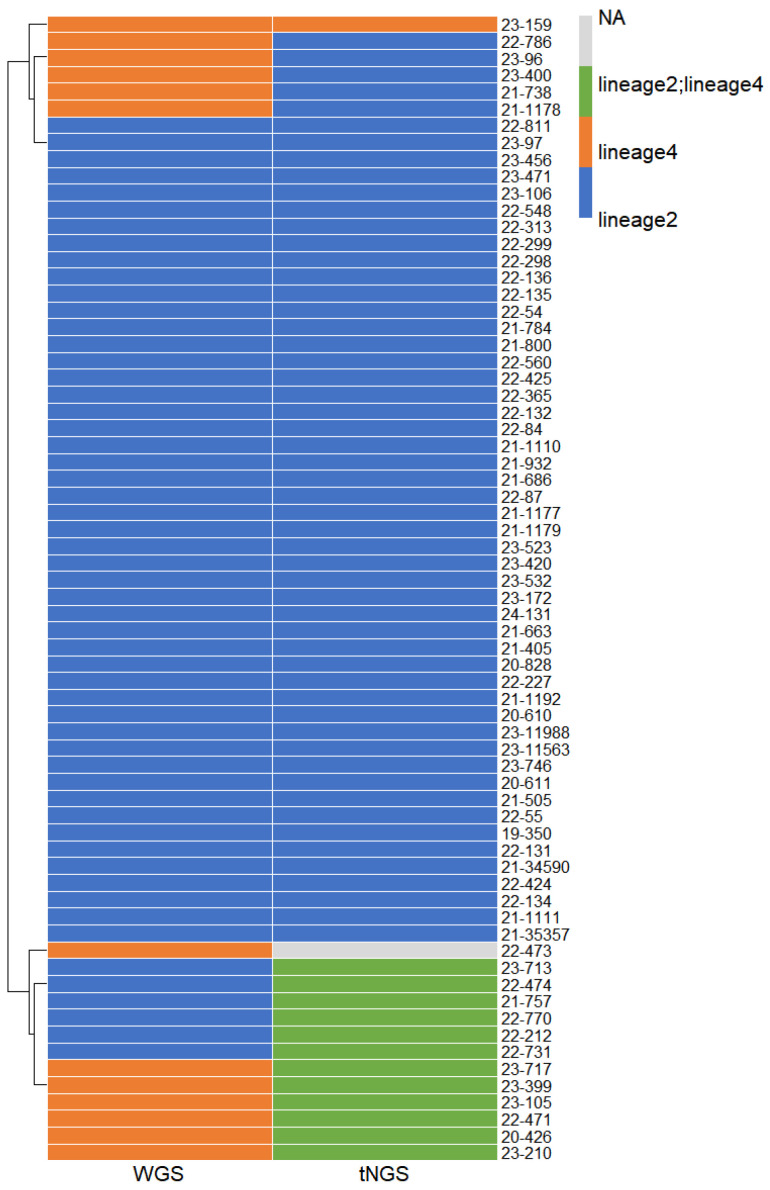
Genotypes of direct sputum tNGS and WGS of cultured isolated strains. NA: No relevant subtype loci captured.

A lineage analysis of M. tuberculosis was performed using tNGS (direct sputum) and WGS (on cultured isolates) (Figure 1). Among the 68 specimens, 50 (73.53%) showed completely concordant lineage results; 12 (17.65%) specimens were identified by tNGS as containing two lineages (lineage 2 and lineage 4), while WGS detected only 1 of them; and 6 (8.82%) specimens were completely discordant, of which 5 were lineage 2 by tNGS but lineage 4 by WGS, and 1 had no lineage call by tNGS.

These results indicate that direct sputum tNGS can detect more complex single-nucleotide polymorphisms, suggesting the presence of mixed infections at the time of initial infection, whereas WGS from cultured isolates provides a more precise determination of the dominant strain’s lineage after culture.

### 2.5. No Difference Between tNGS and WGS When Applied to the Same Cultured Isolate

To further investigate whether the discrepancies between direct sputum tNGS and isolate-based WGS originated from methodological instability, we performed both tNGS and WGS on 24 cultured isolates and compared mutation differences as well as resistance predictions for 14 anti-tuberculosis drugs (Table S2). The results showed that the resistance mutation profiles obtained by tNGS on these isolates were completely identical to those obtained by WGS on the same isolates. This finding demonstrates that, when the two sequencing methods are applied to the same culture material, they yield fully concordant results. Culture-based methods—whether tNGS or WGS—produce highly consistent variant detection. These data confirm that the observed differences in resistance-associated mutations and resistance predictions between direct sputum tNGS and WGS on cultured isolates are attributable to the culture process of mycobacteria from sputum, not to inherent differences in the sequencing technologies themselves. Culture-free sputum tNGS captures the more complex and comprehensive resistance mutation spectrum present in the clinical specimen, including subpopulations that may be amplified, suppressed, or completely lost during in vitro culture expansion.

## 3. Discussion

This study systematically compared sputum-based tNGS with culture-isolate WGS for drug resistance prediction, using pDST as the reference. In agreement with de Diego Fuertes et al. [14], who reported >92% concordance across drugs in rifampicin-resistant patients, and Rosendal et al. [15], who demonstrated the feasibility of direct tNGS on smear-positive specimens, 51.47% of our cases showed fully concordant resistance profiles. We extend these findings by dissecting drug-specific performance and providing direct evidence that culture-driven clonal selection—as documented by Qu et al. [16] through deep amplicon sequencing—is the principal source of discordance.

The decisive proof emerged from the ancillary analysis of 24 cultured isolates: when the same DNA was tested by tNGS and WGS, resistance mutation profiles were completely identical. This mirrors the observation of Zhang et al. [17], who reported full agreement between nanopore-based culture-free tNGS and Illumina WGS, confirming that discrepancies observed in routine workflows reflect genuine biological differences between the in vivo population and the post-culture population. In our cohort, 49 variants were detected exclusively by sputum tNGS versus 25 solely by isolate WGS—a nearly 2:1 ratio—indicating that culture-based strategies systematically underestimate resistance prevalence, echoing the variant gain-and-loss phenomenon described by Qu et al. [16].

Drug-specific analysis revealed a sensitivity–specificity trade-off shaped by this culture bias. For rifampicin, tNGS showed high sensitivity (90.91%) but lower specificity (65.22%) compared to culture-based methods. Crucially, pDST and WGS both rely on the same cultured isolate—a single colony expanded in vitro—whereas tNGS is performed directly on raw sputum, preserving the original within-host diversity, including mixed infections and low-abundance subpopulations that may be lost during culture. This fundamental difference explains why tNGS detects resistant variants that are absent from the matched cultured isolate: culture selects a single dominant clone, especially when the patient harbors multiple strains or a minority resistant subpopulation. Consequently, discordance does not necessarily reflect false-positive tNGS results but rather the inability of culture-based methods (both pDST and WGS) to capture the full complexity of the infecting bacterial population. Direct sputum tNGS, by bypassing culture, provides a more comprehensive resistance profile at the cost of lower concordance with pDST when the latter is treated as an imperfect reference. Indeed, the lower sensitivity but higher specificity of isolate-based WGS compared to direct tNGS stems from this very trade-off: WGS and pDST share the same cultured material; thus, they inherently agree with each other, but both may miss resistance present only in uncultured sputum subpopulations. Moreover, for RIF, tNGS sensitivity was highest (90.91%) by capturing low-abundance resistant subpopulations, but specificity dropped to 65.22%, partly because ten false-positive calls harbored mutations classified as “variable resistance” in the WHO mutation catalogue—an issue addressed by the tiered confidence framework recommended in WHO guidance [18]. INH showed a similar pattern. For the FQs MFX and LFX, however, culture-based WGS offered superior sensitivity and specificity, underscoring its reliability for these critical drugs. EMB exhibited the greatest tNGS sensitivity gain (80.00% vs. 40.00%), likely because its targeted panel captures resistance-conferring loci that do not confer a growth advantage during culture. PZA remained challenging for both methods. The pDST for pyrazinamide itself has major, well-documented reproducibility challenges, with high false-positive resistance rates arising from the acidic culture conditions (pH 5.5) and the use of excessively high inoculum; these challenges may substantially contribute to the observed discordance. Second-line agents (BDQ, DLM, LZD) were fully concordant between tNGS and WGS, yet agreement with pDST varied, reinforcing the WHO guidance that pDST alone may be an imperfect reference for these newer drugs [18].

Phylogenetic resolution was moderate, with tNGS detecting mixed-lineage infections missed by WGS, consistent with the comparative analysis by de Diego Fuertes et al. [14]. Although this does not impair clinical resistance prediction, WGS remains preferable for transmission surveillance. Overall, our results support the WHO-endorsed tiered diagnostic strategy in which sputum tNGS bridges rapid molecular diagnostics and pDST by providing comprehensive resistance profiling, while WGS is reserved for discrepancy resolution and epidemiological investigations. Direct tNGS offers faster results and captures fuller within-host diversity, but its reduced specificity for certain drugs demands cautious interpretation aligned with the graded confidence assigned to each mutation in the WHO catalogue.

This study is limited by its moderate sample size and types, and well-known pDST reproducibility issues for ethambutol and pyrazinamide. The clinical significance of tNGS-exclusive low-frequency variants requires functional and outcome-based validation, as longitudinal clinical data are lacking. Although identical results were obtained from 24 cultured isolates analyzed by both tNGS and WGS—arguing against sequencing depth as the primary cause of discordance—the small number of such isolates limits the definitive attribution of discrepancies to biological differences between sputum and cultured isolates.

In conclusion, sputum tNGS and culture-based WGS show substantial agreement, yet each offers distinct advantages. Sputum tNGS detects mixed infections and low-frequency resistances that are often missed by culture, while WGS provides higher specificity, superior phylogenetic resolution, and more reliable FQ resistance prediction. The culture-dependent clonal selection we and Qu et al. [16] have characterized reveals that culture bias, not technical noise, is the chief source of discordance—an insight that should inform diagnostic algorithms seeking to capture the true landscape of drug-resistant tuberculosis.

## 4. Materials and Methods

### 4.1. Study Design and Human-Subject Research Approval

This retrospective diagnostic accuracy study compared direct sputum tNGS, WGS, and Xpert MTB/RIF for drug resistance prediction in pulmonary tuberculosis, with pDST as the reference standard method. This study was approved by the Ethics Review Committee of the Beijing Center for Disease Control (BJCDC2025017).

### 4.2. Study Population and Specimen Collection

We enrolled 68 consecutive culture-positive pulmonary tuberculosis patients (adults aged ≥ 18 years with no prior anti-TB therapy). A single sputum specimen per patient was divided into three aliquots: one for Xpert MTB/RIF and tNGS, one for culture/pDST, and one for culture-based WGS. An additional 24 cultured isolates were used for method comparison.

### 4.3. Xpert MTB/RIF Assay

Direct sputum specimen was tested using Xpert MTB/RIF (Cepheid, Sunnyvale, CA, USA) per the manufacturer’s instructions. Results were reported as MTB detected (with or without RR) or not detected, with semi-quantitative bacterial load derived from cycle threshold (Ct) values. Ct values were inversely correlated with bacterial load, with semiquantitative categories defined as follows: high (Ct < 16), medium (Ct 16–22), low (Ct 22–28), and very low (Ct > 28).

### 4.4. Direct Sputum tNGS

We extracted DNA from sputum specimens using the Sputum Bacterial DNA Extraction Kit (XP3210-02, Shengshizhongfang (Beijing) Bio Sci & Tech. Co., Ltd., Beijing, China) in accordance with the manufacturer’s operating procedures. Subsequently, the TB Targeted Drug Resistance Detection Kit (XP3170-03, Shengshizhongfang (Beijing) Bio Sci & Tech. Co., Ltd., Beijing, China) was used for library construction, and the length of the obtained library fragments was approximately 280–400 base pairs (bp). An Agilent 2100 Bioanalyzer (Agilent Technologies, Santa Clara, CA, USA) was used to perform the quality control detection of DNA concentration and fragment length. Qualified libraries were mixed and diluted to 2 nM, then subjected to 150 bp paired-end sequencing on the Illumina NovaSeq 6000 sequencing platform (Illumina, San Diego, CA, USA). A high-resolution imaging system was used to collect optical signals, and the digital information was decoded into DNA sequence data. FASTP (version 0.23.2) software was used for the preliminary quality assessment of sequencing reads, BWA (version 0.7.17) was used to align with the laboratory reference strain H37Rv, and Samtools (version 0.1.19) was used for sorting, markup, and indexing. We define a mutation detection threshold to identify single-strand mutations in amplicon sequencing data. Mutations identified at target sites were compared with databases of known drug resistance-related and sublineage-related mutations. Low-frequency variants were screened with set thresholds of ≥20% alternative allele frequency and 20 read counts.

### 4.5. Mycobacterial Culture and pDST

Sputum was decontaminated using N-acetyl-L-cysteine-sodium hydroxide (NALC-NaOH) and cultured on Löwenstein–Jensen and Mycobacterial Growth Indicator Tube (MGIT) 960. We subcultured the sputum specimen in solid medium Löwenstein–Jensen (LJ) and Middlebrooke 7H9 liquid medium MGIT. Positive cultures were confirmed by Ziehl–Neelsen or MPB64 antigen detection. For pDST, isolates were subcultured on Löwenstein–Jensen for 3 weeks. Broth microdilution (MTB drug susceptibility test kit manufactured by Baso Diagnostics Inc., Zhuhai, China) determined minimum inhibitory concentrations (MICs) for 14 drugs (RIF, INH, EMB, PZA, levofloxacin (LFX), moxifloxacin (MFX), bedaquiline (BDQ), delamanid (DLM), linezolid (LZD), streptomycin (STM), amikacin (AMK), kanamycin (KAN), capreomycin CAP, para-aminosalicylic acid (PAS)). MIC was defined as the lowest concentration with no visible growth (Vizion™, Cepheid, Sunnyvale, CA, USA). H37Rv served as quality control. WHO breakpoints: RIF 1 μg/mL, INH 0.12 μg/mL, EMB 4 μg/mL, PZA 100 μg/mL. EMB MIC of 4 μg/mL was considered indeterminate.

### 4.6. WGS on Cultured Isolates

DNA was extracted from pure mycobacterial cultures on Löwenstein–Jensen medium using the Van Embden method [19], and was used for WGS and gDST [11]. Libraries were sequenced on the Illumina NovaSeq 6000 platform. Raw sequencing reads were quality-controlled using fastp to remove low-quality reads, adapter sequences, and sequences containing N bases. If the Q30 of a sample is lower than 80, the sample is deemed unqualified. Reads were mapped to the MTB reference strain H37Rv (NC_000962.3) using the BWA software (version 0.7.17). Variant sites were detected using Freebayes (version 1.3.6). To exclude sequencing errors and false positives, strict filtering thresholds were set: reference genome coverage > 95%, average sequencing depth > 20×, and only variant sites with an alternative allele frequency ≥ 10% were retained. Variants associated with drug resistance were annotated based on the WHO Catalogue of Mycobacterial Resistance Mutations, and resistance prediction for 14 anti-tuberculosis drugs and strain lineage typing were completed.

### 4.7. Direct Comparison of tNGS and WGS on the Same Cultured Isolates

Both methods were applied to 24 cultured isolates using DNA from the same Löwenstein–Jensen culture. tNGS used the 20% allele frequency threshold; WGS used the 10% threshold. Resistance profiles for 14 drugs were compared.

### 4.8. Statistical Analysis

Sensitivity, specificity, and overall agreement with 95% confidence intervals (efficient score method with continuity correction) were calculated using SPSS 22.0.

### 4.9. Data Availability

Raw sequencing data are deposited in Genome Sequence Archive under BioProject accession PRJCA062740.

## Figures and Tables

**Table 1:** Diagnostic performance of Xpert MTB/RIF/tNGS/WGS for RIF compared with pDST.

Method		pDST Resistant	pDST Susceptible	Sensitivity % [95% CI]	Specificity % [95% CI]	Consistency %	Kappa	χ^2^
Xpert MTB/RIF	R	19	9	86.36 (64.04–96.41)	80.43 (65.62–90.14)	82.35	0.624	27.461
	S	3	37					
tNGS	R	20	16	90.91 (69.38–98.41)	65.22 (49.68–78.23)	73.53	0.481	18.818
	S	2	30					
WGS	R	19	6	86.36 (64.04–96.41)	86.96 (73.05–94.58)	86.76	0.708	34.413
	S	3	40					

**Table 2:** Diagnostic performance of tNGS/WGS for other drugs compared with pDST.

Drugs	Method	Sensitivity % [95% CI]	Specificity % [95% CI]	Consistency %
INH	tNGS	82.61 (60.45–94.28)	75.56 (60.14–86.61)	77.94
	WGS	78.26 (55.79–91.71)	91.11 (77.87–97.11)	86.76
EMB	tNGS	80.00 (29.88–98.95)	84.13 (72.28–91.72)	83.82
	WGS	40.00 (7.26–82.96)	85.71 (74.10–92.86)	82.35
PZA	tNGS	9.30 (3.02–23.05)	88.00 (67.66–96.85)	38.24
	WGS	13.95 (5.80–28.63)	88.00 (67.66–96.85)	41.18
MFX	tNGS	50.00 (29.65–70.35)	86.36 (71.95–94.33)	73.53
	WGS	70.83 (48.75–86.56)	97.73 (86.49–99.88)	88.24
LFX	tNGS	58.82 (33.45–80.57)	84.31 (70.86–92.52)	77.94
	WGS	76.47 (49.76–92.18)	90.20 (77.81–96.33)	86.76
BDQ	tNGS	0.00 (0.00–80.21)	100.00 (93.15–100.00)	97.06
	WGS	0.00 (0.00–80.21)	100.00 (93.15–100.00)	97.06
DLM	tNGS	0.00 (0.00–19.24)	100.00 (90.59–100.00)	69.12
	WGS	0.00 (0.00–19.24)	100.00 (90.59–100.00)	69.12
LZD	tNGS	0.00 (0.00–60.42)	100.00 (92.95–100.00)	94.12
	WGS	0.00 (0.00–60.42)	100.00 (92.95–100.00)	94.12
STM	tNGS	80.00 (58.70–92.39)	81.40 (66.08–91.08)	80.88
	WGS	76.00 (54.48–89.84)	95.35 (82.94–99.19)	88.24
AMK	tNGS	0.00 (0.00–60.42)	100.00 (92.95–100.00)	94.12
	WGS	0.00 (0.00–60.42)	100.00 (92.95–100.00)	94.12
KAN	tNGS	0.00 (0.00–43.91)	100.00 (92.62–100.00)	89.71
	WGS	0.00 (0.00–43.91)	100.00 (92.62–100.00)	89.71
CAP	tNGS	0.00 (0.00–32.14)	100.00 (92.13–100.00)	83.82
	tNGS	0.00 (0.00–32.14)	100.00 (92.13–100.00)	83.82
PAS	WGS	0.00 (0.00–13.34)	100.00 (87.99–100.00)	52.94
	tNGS	0.00 (0.00–13.34)	100.00 (87.99–100.00)	52.94

## Data Availability

The raw sequence data reported in this paper have been deposited in the Genome Sequence Archive (Genomics, Proteomics & Bioinformatics 2025 [20]) in National Genomics Data Center (Nucleic Acids Res 2026 [21]), China National Center for Bioinformation/Beijing Institute of Genomics, Chinese Academy of Sciences (GSA: CRA041904; CRA042017; CRA042018) that are publicly accessible at https://ngdc.cncb.ac.cn/gsa (accessed on 21 April 2026).

## Supplementary Materials

The following supporting information can be downloaded at: https://www.mdpi.com/article/10.3390/ijms27125392/s1.
